# Attenuated TGFB signalling in macrophages decreases susceptibility to DMBA-induced mammary cancer in mice

**DOI:** 10.1186/s13058-021-01417-8

**Published:** 2021-03-24

**Authors:** Xuan Sun, Sarah M. Bernhardt, Danielle J. Glynn, Leigh J. Hodson, Lucy Woolford, Andreas Evdokiou, Cong Yan, Hong Du, Sarah A. Robertson, Wendy V. Ingman

**Affiliations:** 1 Discipline of Surgery, Adelaide Medical School, The Queen Elizabeth Hospital, University of Adelaide, Adelaide, Australia; 2 Robinson Research Institute, University of Adelaide, Adelaide, Australia; 3 School of Animal and Veterinary Sciences, University of Adelaide, Adelaide, SA Australia; 4 Department of Pathology and Laboratory Medicine, Indiana University, Indianapolis, IN USA; 5 Adelaide Medical School, University of Adelaide, Adelaide, Australia; 6 Discipline of Surgery, The Queen Elizabeth Hospital, DX465702, 28 Woodville Rd., Woodville, 5011 Australia

**Keywords:** Transforming growth factor beta, Mammary gland, Macrophage, Cancer

## Abstract

**Background:**

Transforming growth factor beta1 (TGFB1) is a multi-functional cytokine that regulates mammary gland development and cancer progression through endocrine, paracrine and autocrine mechanisms. TGFB1 also plays roles in tumour development and progression, and its increased expression is associated with an increased breast cancer risk. Macrophages are key target cells for TGFB1 action, also playing crucial roles in tumourigenesis. However, the precise role of TGFB-regulated macrophages in the mammary gland is unclear. This study investigated the effect of attenuated TGFB signalling in macrophages on mammary gland development and mammary cancer susceptibility in mice.

**Methods:**

A transgenic mouse model was generated, wherein a dominant negative TGFB receptor is activated in macrophages, in turn attenuating the TGFB signalling pathway specifically in the macrophage population. The mammary glands were assessed for morphological changes through wholemount and H&E analysis, and the abundance and phenotype of macrophages were analysed through immunohistochemistry. Another cohort of mice received carcinogen 7,12-dimethylbenz(a)anthracene (DMBA), and tumour development was monitored weekly. Human non-neoplastic breast tissue was also immunohistochemically assessed for latent TGFB1 and macrophage marker CD68.

**Results:**

Attenuation of TGFB signalling resulted in an increase in the percentage of alveolar epithelium in the mammary gland at dioestrus and an increase in macrophage abundance. The phenotype of macrophages was also altered, with inflammatory macrophage markers iNOS and CCR7 increased by 110% and 40%, respectively. A significant decrease in DMBA-induced mammary tumour incidence and prolonged tumour-free survival in mice with attenuated TGFB signalling were observed. In human non-neoplastic breast tissue, there was a significant inverse relationship between latent TGFB1 protein and CD68-positive macrophages.

**Conclusions:**

TGFB acts on macrophage populations in the mammary gland to reduce their abundance and dampen the inflammatory phenotype. TGFB signalling in macrophages increases mammary cancer susceptibility potentially through suppression of immune surveillance activities of macrophages.

## Introduction

Transforming growth factor beta1 (TGFB1) is a multi-functional cytokine with diverse roles in the regulation of cellular function, including proliferation, differentiation, migration, apoptosis and the immune response[1–3]. In the mammary gland, TGFB1 is predominantly produced by the mammary epithelium although TGFB1 may also come from a variety of other sources such as immune cells and from systemic circulation [4–6]. Many different cell types in the mammary gland are responsive to TGFB including epithelial cells, fibroblasts and immune cells; therefore, it can be difficult to identify the precise cell types and mechanisms through which TGFB1 exerts its many actions.

In the mammary gland, TGFB1 inhibits epithelial cell proliferation, cell death and ductal development [7–11]. TGFB1 is also implicated in tumour development and progression, and both stimulatory and inhibitory roles have been described [12–14]. In the early stages of tumourigenesis, TGFB1 acts as a tumour suppressor, through inhibition of cell proliferation, induction of apoptosis and suppression of growth factor, cytokine and chemokine production. As tumours progress, tumour cells can express abundant TGFB1 which appears to have largely pro-tumorigenic effects. This overexpression of TGFB1 by tumour cells modulates epithelial-mesenchymal transition, impairs immune surveillance and promotes angiogenesis, therefore promoting tumour invasion and metastasis.

Macrophages are bone marrow-derived cells present in the mammary gland stroma and regulate epithelial cell function during normal mammary gland development [15–17]. In breast cancer, macrophages play multiple roles in metastasis, angiogenesis and cell invasion and promote the growth and survival of tumour cells [18–20]. Macrophages are key target cells for TGFB1 action. Epithelial cell-derived TGFB1 regulates macrophage function and phenotype in the mammary gland [21], and macrophages may act as intermediary cells through which TGFB1 affects tumourigenesis. However, the impact of TGFB signalling on local macrophage populations in the mammary gland is still unclear.

TGFB1 signalling has also been implicated in susceptibility to breast cancer, where an association between breast cancer risk and the TGFB1 L10P gene polymorphism was reported by the Breast Cancer Association Consortium [22]. The TGFB1 L10P gene is linked to the increased cellular expression of TGFB1 [23], suggesting that increased abundance of TGFB1 might increase breast cancer risk. However, as TGFB1 is known as both a suppressor and promoter of breast cancer, the precise mechanisms to which it contributes to cancer risk remain unclear. Given the role of TGFB1 in regulating macrophage phenotype and function, we proposed that TGFB-regulated macrophages could affect breast cancer susceptibility.

In this study, a double transgenic mouse model was developed by breeding *Cfms-rtTA* and *TetO-TbrII* single transgenic mice. In the double transgenic mouse model, a dominant negative TGFB receptor is expressed specifically in macrophages in the presence of doxycycline. The expression of the dominant negative receptor attenuates TGFB signalling specifically in the macrophage population. The two single transgenic mouse strains used to generate the double transgenic were used as controls, and all mice were given doxycycline in their drinking water. The impact of attenuated TGFB signalling in macrophages on mammary gland development and mammary cancer susceptibility was investigated.

## Methods

### Animals

All animal experiments were approved by the University of Adelaide Animal Ethics Committee and were conducted in accordance with the Australian Code of Practice for the Care and Use of Animals for Scientific Purposes [24]. All mice were maintained in specific pathogen-free conditions with controlled light (12-h light, 12-h dark cycle) and temperature at the Laboratory Animal Services Medical School facility. Doxycycline-treated mice received doxycycline (2 mg/mL; Sigma-Aldrich) in drinking water from the age of 6 weeks until culled.

*Cfms-rtTA* transgenic mice [25] were imported from Indiana University, USA, from Prof. Cong Yan’s laboratory. In this transgenic mouse model, the expression of a reverse tetracycline/doxycycline-responsive transactivator (rtTA) is driven by the macrophage-specific *Cfms* promoter such that *rtTA* expression is restricted to macrophages and is induced by administration of doxycycline.

*TetO-TbrII* transgenic mice [26] were purchased from the Jackson Laboratories (ME, USA). The disrupted *TbrII* gene encodes a dominant negative TGFB receptor which results in disrupted TGFB signalling. The expression of the dominant negative TGFB receptor is under the control of the *TetO* promoter. However, the transgene is silent in *TetO-TbrII* mice as the *TetO* promoter only drives the expression after activation by a specific tetracycline/doxycycline-responsive transactivator.

*Cfms-TbrII* double transgenic mice were generated by crossbreeding of *Cfms-TbrII* and *TetO-TbrII* single transgenic mice such that *Cfms-TbrII* mice carried one allele for each of the two transgenes. In the *Cfms-TbrII* double transgenic mouse, the expression of the transgenes is induced by the addition of doxycycline.

To track the oestrous cycle stage, analysis of vaginal smears was performed as described previously [27, 28]. The oestrous cycles of mice were tracked daily for at least 28 days.

### RNA extraction and quantitative real-time PCR

Total RNA was extracted from snap-frozen tissue dissected from *Cfms-rtTA*, *TetO-TbrII* and *Cfms-TbrII* mice, using TRIzol (Invitrogen, CA, USA). Extracted RNA was treated with DNase to remove contaminating DNA, using reagents supplied in a TURBO DNA-free kit (Life Technologies) according to the manufacturer’s instructions.

Expression of mRNA encoding the dominant negative TGFB receptor (*ΔTgfbrII*) was quantified through quantitative real-time PCR (RT-PCR). RT-PCR amplification was performed using an ABI Prism 7000 Sequence Detection System with either a TaqMan Universal MasterMix II (Life Technologies) or a 2×SYBR Green PCR Master Mix (Life Technologies) according to the manufacturer’s instructions. Primer pairs specific for dominant negative TGFB receptor (Genbank accession number NM_029575.3) mRNA expression were designed and custom made by Life Technologies (Custom Plus TaqMan® RNA Assay) and supplied as a single tube that contains primers and probe. The *ΔTgfbrII* assay ID was AJ89J6H and produced an amplicon of 79 bp. Expression of the *ΔTgfbrII* transgene was measured in duplicate and normalised to the house-keeping gene *Actb* (Genbank accession number NM_007393, assay ID Mm00607939_s1; amplicon length 115).

### DMBA-induced mammary cancer

The susceptibility of mice to mammary gland cancer was investigated using the 7,12-dimethylbenz(a)anthracene (DMBA)-induced mammary tumour model. *Cfms-rtTA*, *TetO-TgfbrII* and *Cfms-TbrII* mice received DMBA in sesame oil (1 mg/mL) weekly by oral gavage for 6 weeks from 6 weeks of age. Following the final carcinogen treatment, mice were palpated for tumours weekly until detection of a mammary gland tumour. Mice were then euthanised by cervical dislocation, and tumours were dissected for further analysis. Lesions were assessed in haematoxylin and eosin (H&E)-stained sections by veterinary pathologist LW to confirm the presence of mammary gland cancer.

### Tissue morphology and pathology

To investigate the effect of attenuated TGFB signalling in macrophages on mammary gland development at dioestrus, one side of the 4th pair of mammary glands from 3-month-old virgin doxycycline-treated *Cfms-rtTA*, *TetO-TbrII* and *Cfms-TbrII* females was dissected and stained as a whole mount with carmine alum as previously described [21]. The other side of the 4th pair of the mammary gland was paraffin-embedded, sectioned and stained with H&E. The 3rd pair of mammary glands were also dissected and fresh frozen in OCT.

Whole-mount images of the mammary glands were captured by MZ16 FA-Stereo microscope (Leica, The University of Adelaide, SA, Australia). H&E sections were captured as a digital image using a Nanozoomer 1.0 (Hamamatsu, Shizuoka, Japan) at a zoom equivalent to a × 40 objective lens. Quantification analysis of wholemounts, histology, immunohistochemistry and immunofluorescence was performed by an assessor blinded to mouse genotype. To determine the extent of ductal branching morphogenesis in the whole-mounted mammary glands, the three longest ducts from each mammary gland were selected, and the number of branch points on each duct was counted manually and expressed as branch points/mm.

To quantify the extent of alveolar development in the mammary gland, the epithelium was categorised as ductal (single epithelium layer) or alveolar (clusters of epithelial structures containing alveolar lumens). The classification of the ductal and alveolar epithelium was based on that developed by Fata et al. [29] and has been used previously [21, 30]. Ductal epithelium was defined as a single epithelial cell layer, and alveolar epithelium was defined as clusters of epithelial structures containing alveolar lumens (analogous to grades 0 and 3, respectively, according to the method developed by Fata et al.). The number of the ductal and alveolar epithelial structures were counted manually in H&E-stained sections, and one full section that included the inguinal lymph node (as a marker for the mid-section of the gland) quantified per mouse. The total number of alveolar structures in the section was expressed as a percentage of total epithelial structures (% alveolar structures = 100 × (alveolar structures/(ductal structures + alveolar structures)).

### Immunohistochemistry

Macrophage abundance was determined by F4/80 antibody staining, using rat anti-F4/80 monoclonal antibody (1:50 dilution; overnight at 4 °C) (Caltag Laboratories, Burlingame, CA, USA), followed by biotinylated rabbit anti-rat IgG (1:100 dilution; 40 min at room temperature) (Vector Laboratories) and ABC Elite kit (Vector Laboratories) with 3,3 diaminobenzadine (DAB) peroxidase (DAKO, Denmark).

CCR7 was detected using rat anti-CCR7 monoclonal antibody (1:200 dilution; overnight at 4 °C) (Abnova), followed by rabbit anti-rat HRP (1:200; 60 min at room temperature) (Dako). The detection of bound antibody was performed using DAB (DAKO, Denmark) according to the manufacturer’s instructions.

iNOS was detected on fresh frozen tissue sections from OCT-embedded tissue using rabbit anti-iNOS polyclonal antibody (1:300 dilution, overnight at 4 °C) (BD Pharmingen, BD Biosciences, San Diego, USA), followed by goat anti-rabbit HRP (1:400; 40 min at room temperature), and detected with using DAB peroxidase (DAKO, Denmark).

The stained tissue sections were captured as a digital image using a Nanozoomer at a zoom equivalent to a × 40 objective lens. The number of F4/80-, CCR7- and iNOS-positive cells in the mammary gland was counted, where only positive cells with visible haematoxylin-stained nuclei were included. For F4/80 staining, F4/80-positive macrophages were distinguished from F4/80-stained eosinophils on the basis of nuclear morphology. All results were expressed as positive cells/mm^2^. The mean density of positive cells within the five ductal and the five alveolar stroma was calculated and grouped by mouse genotype.

### Immunofluorescence

Macrophages positive for phosphorylated SMAD2, indicative of TGFB signalling [31], were detected by immunofluorescent staining of the formalin-fixed paraffin-embedded mammary glands. Following antigen retrieval, F4/80 antibody conjugated to Alexa-750 (Caltag Laboratories, Burlingame, CA, USA) and rabbit anti-pSMAD2 monoclonal antibody (Invitrogen) both at a 1:100 dilution were incubated with the slides overnight at 4 °C. Goat anti-rabbit antibody conjugated to Alexa-488 was used to detect pSMAD2 (1:500 dilution; 1 h at 4 °C) followed by 4′, 6-diamidino-2-phenylindol (DAPI) (Sigma, St Louis, USA) staining for 10 min at 1:1000 dilution. Images were captured and collected using an Olympus FV3000 Confocal Microscope at × 40 magnification. Macrophages were selected randomly for imaging, and the number of macrophages positive for pSMAD2 quantified and expressed as percent positive per mammary gland.

### Human non-neoplastic breast tissue

Human non-neoplastic breast tissue was collected from female patients undergoing either reduction mammoplasty or mastectomy surgery at The Queen Elizabeth Hospital. Tissue collection was approved by the Human Research Ethics Committee at the University of Adelaide and The Queen Elizabeth Hospital (TQEH Ethics Approval #2011120). Participants were included if they were a woman aged between 18 and 75 years of age and capable of giving informed consent. Participants were excluded from the study in the event of pregnancy, current chemotherapy or high dependence on medical care resulting in the inability to give informed consent.

Macrophages were detected using monoclonal mouse anti-human CD68 primary antibody (1:50 dilution; 30 min) (Dako, Denmark). Before the sections were incubated with the primary antibody, the sections were placed in Dako EnVision™ high pH antigen retrieval solution (Dako) and brought to 90 °C in a water bath for 20 min. After primary antibody incubation, the sections were incubated with Dako EnVision™ HRP (ready-to-use, Dako) for 30 min at room temperature, and the detection of bound antibody was performed using DAB according to the manufacturer’s instructions. The tissue sections were counterstained with haematoxylin prior to dehydration and mounting. Mouse anti-human IgG was used as an isotype control. Stained tissue sections were captured as a digital image using a Nanozoomer at a zoom equivalent to a × 40 objective lens. Three epithelium clusters were randomly selected from each section for quantification. For each epithelium cluster, the total area of the cluster (mm^2^), the area of the epithelium (mm^2^) and the area of the stroma (mm^2^) were measured and calculated. The number of CD68-positive cells in each cluster was counted manually, and only positive cells with visible haematoxylin-stained nuclei were included. All results were expressed as positive cells/mm^2^. The mean density of positive cells within the cluster’s epithelium and stroma from each patient was calculated.

TGFB1 was detected through immunofluorescence using polyclonal affinity-purified chicken anti-latent TGFB1 (1:40 dilution; overnight at 4 °C) (R&D Systems, MN, USA), followed by anti-chicken IgY FITC-conjugated secondary antibody (1:200 dilution; 60 min at room temperature) (Sigma-Aldrich). The sections were mounted in a fluorescent mounting medium (Dako, Glostrup, Denmark) with DAPI and were stored at 4 °C in the dark until image capture. Fluorescence images of TGFB1 staining were captured and collected using FV10i Confocal Microscope (Olympus, USA) with laser-power and photomultiplier settings kept constant for all experiments, and images were captured at × 60 magnification. Three epithelium clusters were randomly selected from each section. Using the ImageJ software, the mean staining intensity of TGFB1 within epithelium was determined, and a mean value of 3 clusters per patient was calculated.

### Statistical analysis

Data were assessed for normal distribution with a Shapiro-Wilk normality test using GraphPad Prism 8 (GraphPad Software Inc., San Diego, USA) or SPSS Statistics version 17.0 (IBM Corporation, Armonk, NY, USA) and presented as mean ± standard error of the mean (s.e.m.). Differences between the means were evaluated by a one-way ANOVA with post hoc multiple comparisons test (Tukey’s multiple comparison test) if data were normally distributed. The non-parametric Kruskal-Wallis test (with Dunn’s multiple comparison’s analysis) was used if data were not normally distributed. Linear regression analysis was conducted using SPSS Statistics version 17.0 (IBM Corporation, Armonk, NY, USA) if analysis of relationships between variables was required. Kaplan-Meier survival curves were generated using SPSS Statistics version 17.0 to analyse the survival function, and the log rank test was used to compare different Kaplan-Meier curves between the groups. The difference between the groups was considered statistically significant if *p* < 0.05.

## Results

### Reduced positivity of macrophages for phosphorylated SMAD2 and elevated expression of the dominant negative TGFB receptor transgene in *Cfms-TbrII* mice

**Fig. 1:**
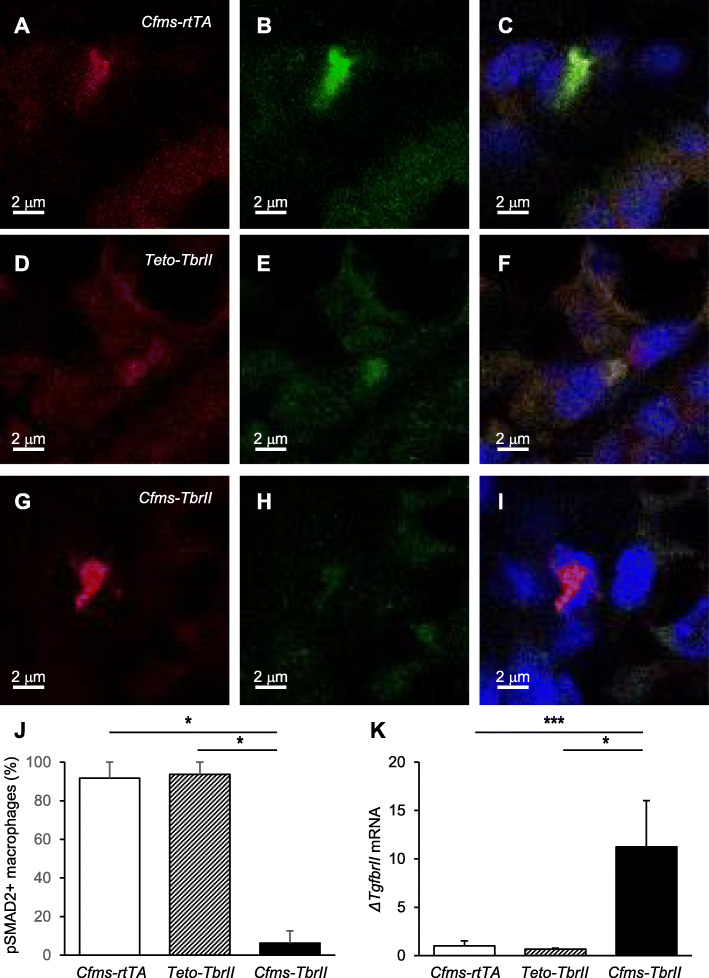
Attenuation of TGFB signalling in macrophages from Cfms-TbrII transgenic mice. Representative immunofluorescent staining of the mammary glands with F4/80 (red channel; **a**, **d**, **g**) and pSMAD2 (green channel; **b**, **e**, **h**) antibodies, and merged images showing co-localisation and DAPI (blue channel) nuclear stain (**c**, **f**, **i**) from single transgenic control *Cfms-rtTA* (**a**–**c**) mice, *TetO-TbrII* (**d**–**f**) mice and double transgenic *Cfms-TbrII* (**g**–**i**) mice. Co-localisation was quantified and expressed as a percent of F4/80-positive macrophages that are pSMAD2 positive (*n* = 4/gp; **j**). The spleens collected from doxycycline-treated single transgenic control *Cfms-rtTA*, *TetO-TbrII* and double transgenic *Cfms-TbrII* mice, were analysed for *ΔTgfbrII* mRNA by RT-PCR and normalised to *Actb* expression (*n* = 12–13/gp; **k**). The result is given in arbitrary units where the average of the single transgenic control *Cfms-rtTA* is 1. Data are presented as mean + SEM with statistical analysis using a Kruskal-Wallis test, *p < 0.05, ***p < 0.005

To determine whether doxycycline-treated *Cfms-TbrII* mice exhibited attenuated TGFB signalling in macrophages, co-localisation of F4/80 and pSMAD2 was investigated. Macrophages detected in single transgenic control doxycycline-treated *Cfms-rtTA* and *TetO-TbrII* mice exhibited co-localisation with pSMAD2 (1a-c and 1d-f, respectively) whilst there was little co-localisation detected in *Cfms-TbrII* mice (Fig. 1g–i). Single-stained controls exhibited little to no staining in the green channel (F4/80 only) or red channel (pSMAD2 only) (Supplementary Figure S1) There were significantly less pSMAD-positive macrophages in *Cfms-TbrII* mice compared to *Cfms-rtTA* and *TetO-TbrII* controls (Fig. 1j). Messenger RNA expression of the dominant negative TGFB receptor *ΔTgfbrII* was quantified in the spleens of doxycycline-treated transgenic mice by RT-PCR (Fig. 1k). The expression of *ΔTgfbrII* mRNA was significantly higher in doxycycline-treated *Cfms-TbrII* mice compared to doxycycline-treated *Cfms-rtTA* and *TetO-TbrII* controls. These results confirmed that the dominant negative TGFB receptor transgene is specifically expressed by doxycycline-treated *Cfms-TbrII* double transgenic mice and results in attenuated TGFB signalling in mammary gland macrophages.

### Effect of attenuated TGFB signalling in macrophages on oestrus cyclicity

To investigate the effect of attenuated TGFB signalling on oestrous cyclicity, cycles were tracked for a period of 28 days by histological analysis of vaginal smears in adult females for doxycycline-treated *Cfms-rtTA*, *TetO-TbrII* and *Cfms-TbrII* mice. The oestrous cycle length and the number of cycles within 28 days from each group were unaffected by the mouse genotype (Table 1). There was no significant difference in the percentage of time spent in any stage of the oestrous cycle amongst the three groups.

**Table 1:** The average length of the oestrous cycle and the number of oestrous cycles with 28 days in adult doxycycline-treated *Cfms-rtTA*, *TetO-TbrII* and *Cfms-TbrII* mice. Oestrous cycles were tracked for 28 days (between 8 and 12 weeks of age) by histological analysis of vaginal smears. Oestrus was defined as when > 90% of cells in vaginal smear were cornified epithelial cells. A single complete cycle was defined as the first day of oestrus through to the first day of the next oestrus (*n* = 8–12 per group). Data are presented as mean ± SEM, with statistical analysis conducted using a one-way ANOVA test (no significant differences detected)

	*Cfms-rtTA*	*TetO-TgfbrII*	*Cfms-TbrII*
Cycle length (days)	5.04 ± 0.07	4.93 ± 0.11	4.84 ± 0.10
Cycles (number)	4.8 ± 0.13	4.63 ± 0.11	4.75 ± 0.25

### Attenuated TGFB signalling affects mammary gland development at dioestrus

**Fig. 2:**
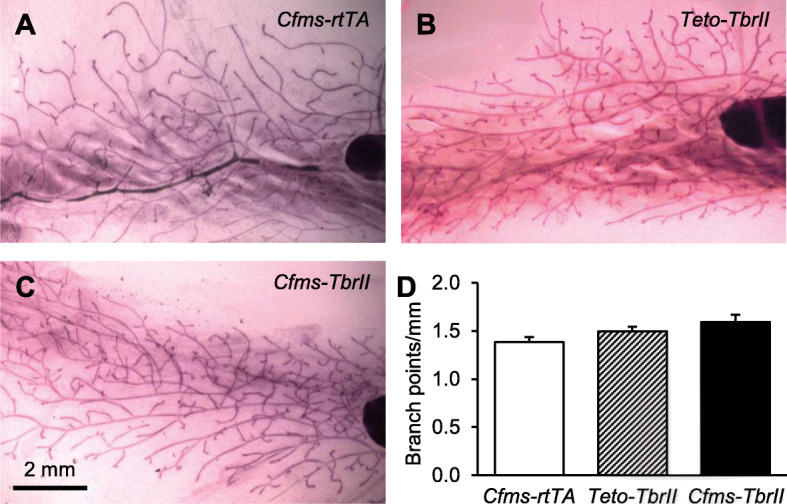
The effect of attenuated TGFB signalling in macrophages on mammary gland morphogenesis. Representative mammary gland carmine alum-stained wholemounts of doxycycline-treated single transgenic control *Cfms-rtTA* (*n* = 10; **a**), *TetO-TbrII* (*n* = 8; **b**) and double transgenic *Cfms-TbrII* (*n* = 12; **c**) mice at dioestrus. The number of branch points/mm was calculated from the analysis of wholemounts (**d**). Data are presented as mean + SEM with statistical analysis using a one-way ANOVA test

**Fig. 3:**
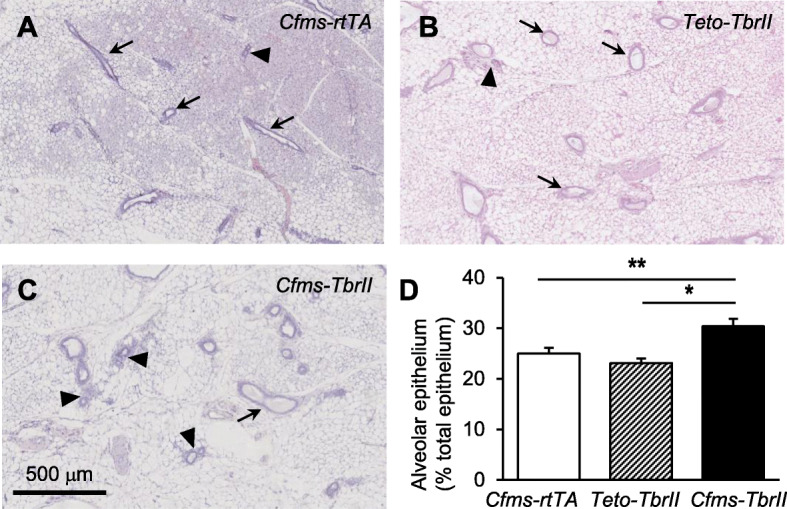
The effect of attenuated TGFB signalling in macrophages on mammary gland morphogenesis. Representative mammary gland H&E-stained sections of doxycycline-treated single transgenic control *Cfms-rtTA* (*n* = 10; **a**), *TetO-TbrII* (*n* = 8; **b**), and double transgenic *Cfms-TbrII* (*n* = 12; **c**) mice at dioestrus. Examples of ductal epithelium are identified by arrows, and examples of alveolar epithelium are identified by arrowheads. The alveolar epithelium was quantified and expressed as a percentage of the total epithelium (**d**). Data are presented as mean + SEM with statistical analysis using a one-way ANOVA test, *p < 0.05, **p < 0.01

Previous studies have shown that both TGFB1 [10] and macrophages [15] are most abundant in the mammary gland at dioestrus; therefore, this stage of the cycle was chosen to examine the effect of TGFB signalling in macrophages on mammary gland development. The overt morphology of mammary gland wholemounts was not affected by the transgene, and branch points per millimetre of the duct were not altered (Fig. 2). However, there was a small but significant increase in the percent of alveolar epithelial buds in mice with attenuated TGFB signalling in macrophages (Fig. 3; 20% increase and 30% increase compared to *Cfms-rtTA* and *TetO-TbrII* controls, respectively). There was no significant difference in the percentage of alveolar epithelium in the mammary glands from *Cfms-rtTA* control mice compared to *TetO-TbrII* control mice.

### Attenuated TGFB signalling increases macrophage abundance and inflammatory macrophage markers

**Fig. 4:**
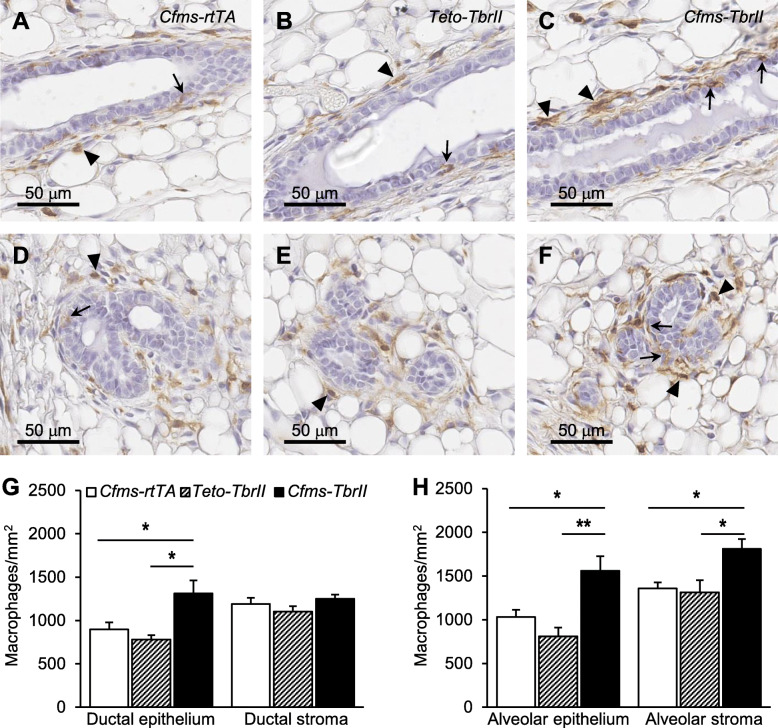
The effect of attenuated TGFB signalling on macrophage abundance and location in the mammary gland. Representative F4/80 antibody staining of macrophages around the ductal (**a**–**c**) and alveolar (**d**–**f**) epithelium. Examples of F4/80-positive epithelial cell-associated macrophages are indicated by arrows, and examples of F4/80-positive stromal macrophages are indicated by arrowheads. The number of F4/80-positive cells within the ductal epithelium and stroma (**g**) and within the alveolar epithelium and stroma (**h**) was calculated in the mammary glands from doxycycline-treated single transgenic control *Cfms-rtTA* (*n* = 10; **a**, **d**) mice, *TetO-TbrII* (*n* = 8; **b**, **e**) mice and double transgenic *Cfms-TbrII* (*n* = 12; **c**, **f**) mice. Data are presented as mean + SEM with statistical analysis using a one-way ANOVA test, *p < 0.05, **p < 0.01

F4/80 is expressed at high levels on the cell membrane of macrophages and is a well-known macrophage differentiation marker [32]. F4/80-positive macrophages were observed in the mammary stroma surrounding the ductal and alveolar epithelium, as well as directly adjacent and in close contact with the epithelium of all transgenic mice (Fig. 4). No positive staining was observed in the mammary epithelium stained with isotype-matched irrelevant antibody (Supplementary Figure S2A). No significant differences were observed in the abundance of macrophages within the stroma or surrounding the ductal or alveolar epithelium between the two single transgenic control groups (Fig. 4g, h). There was a 46% and 68% increase in the number of macrophages within *Cfms-TbrII* ductal epithelium compared to that of *Cfms-rtTA* ductal epithelium and *TetO-TbrII* ductal epithelium, respectively (Fig. 4g). No significant differences in macrophage density within the ductal stroma were observed amongst the three genotypes (Fig. 4g). There was a 51% and 92% increase in the number of macrophages within *Cfms-TbrII* alveolar epithelium, compared to that of *Cfms-rtTA* alveolar epithelium and *TetO-TbrII* alveolar epithelium, respectively (Fig. 4h). Additionally, there was a 33% and 37% increase in the number of macrophages within *Cfms-TbrII* alveolar stroma, compared to that of *Cfms-rtTA* alveolar stroma and *TetO-TbrII* alveolar stroma, respectively (Fig. 4h).

**Fig. 5:**
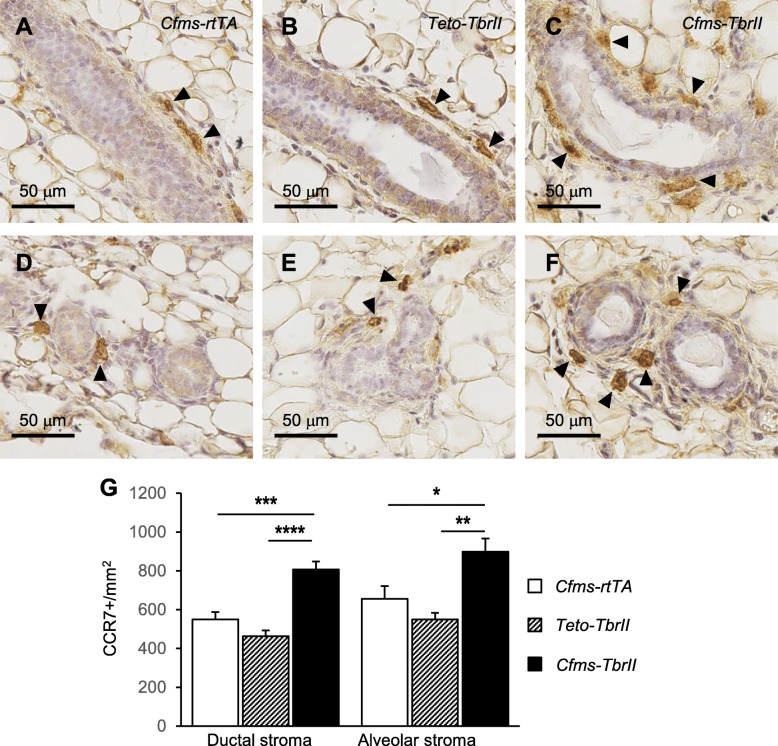
The effect of attenuated TGFB signalling on abundance and location of CCR7-positive cells within the mammary stroma. Representative CCR7 antibody staining in the stroma around the ductal (**a**–**c**) and alveolar (**d**–**f**) epithelium. Examples of CCR7-positive stromal macrophages are indicated by arrowheads. The number of CCR7-positive (**g**) cells within the stroma was calculated in the mammary glands from doxycycline-treated single transgenic control *Cfms-rtTA* (*n* = 10; **a**, **d**) mice, *TetO-TbrII* (*n* = 8, **b**, **e**) mice and double transgenic *Cfms-TbrII* (*n* = 12; **c**, **f**) mice. Data are presented as mean + SEM with statistical analysis using a one-way ANOVA test, *p < 0.05, **p < 0.01, ***p < 0.005, ****p < 0.001

CCR7 is a chemokine receptor involved in lymphocyte homing [33] and is a phenotypic marker of “M1” or inflammatory type macrophages [34, 35]. CCR7-positive cells were located in the stroma surrounding the ductal and alveolar epithelium in the mammary glands of doxycycline-treated *Cfms-rtTA*, *TetO-TbrII* and *Cfms-TbrII* mice (Fig. 5). No positive staining was observed in the mammary epithelium stained with isotype-matched irrelevant antibody (Supplementary Figure S2B). There was a 47% and 74% increase in the number of CCR7-positive cells within *Cfms-TbrII* ductal stroma compared to that of *Cfms-rtTA* ductal stroma and *TetO-TbrII* ductal stroma, respectively (Fig. 5g). There was a 37% and 63% increase in the number of CCR7-positive cells within *Cfms-TbrII* alveolar stroma compared to that of *Cfms-rtTA* alveolar stroma and *TetO-TbrII* alveolar stroma, respectively. No significant differences were detected in the number of CCR7-positive macrophages within the stroma surrounding the ductal or alveolar epithelium between the two control groups.

**Fig. 6:**
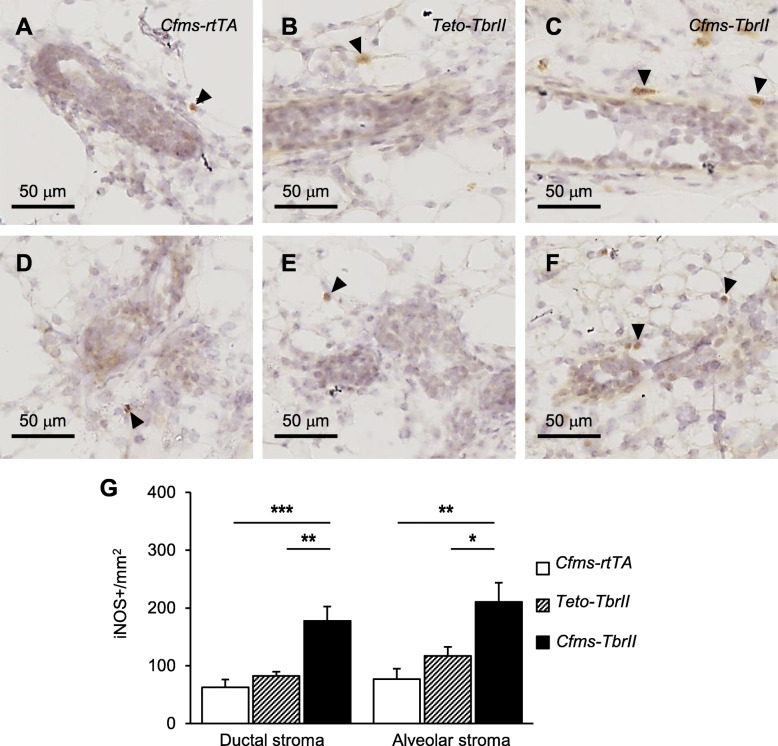
The effect of attenuated TGFB signalling on abundance and location of iNOS-positive cells within the mammary stroma. Representative iNOS antibody staining in the stroma around the ductal (**a**–**c**) and alveolar (**d**–**f**) epithelium. Examples of iNOS-positive stromal macrophages are indicated by arrowheads. The number of iNOS -positive (**g**) cells within the stroma was calculated in the mammary glands from doxycycline-treated single transgenic control *Cfms-rtTA* (*n* = 10; **a**, **d**) mice, *TetO-TbrII* (*n* = 8, **b**, **e**) mice and double transgenic *Cfms-TbrII* (*n* = 12; **c**, **f**) mice. Data are presented as mean + SEM with statistical analysis using a one-way ANOVA test, *p < 0.05, **p < 0.01, ***p < 0.005

iNOS is highly expressed by activated cytotoxic macrophages involved in antigen presentation and immune surveillance [36] and is considered a marker for “M1” or inflammatory type macrophages [34]. iNOS expression was observed in the mammary gland epithelium stromal compartment (Fig. 6). No positive staining was observed in the mammary gland stained with isotype-matched irrelevant antibody (Supplementary Figure S2C). There was a 180% and 110% increase in the number of iNOS-positive macrophages within *Cfms-TbrII* ductal stroma compared to that of *Cfms-rtTA* ductal stroma and *TetO-TbrII* ductal stroma, respectively (Fig. 6g). In addition, there was a 180% and 80% increase in the number of iNOS-positive macrophages within *Cfms-TbrII* alveolar stroma compared to that of *Cfms-rtTA* alveolar stroma and *TetO-TbrII* alveolar stroma, respectively. No significant differences were detected in the number of iNOS-positive macrophages within the stroma surrounding the ductal or alveolar epithelium between the two control groups.

### Attenuated TGFB signalling in macrophages decreases mammary cancer susceptibility in mice

**Fig. 7:**
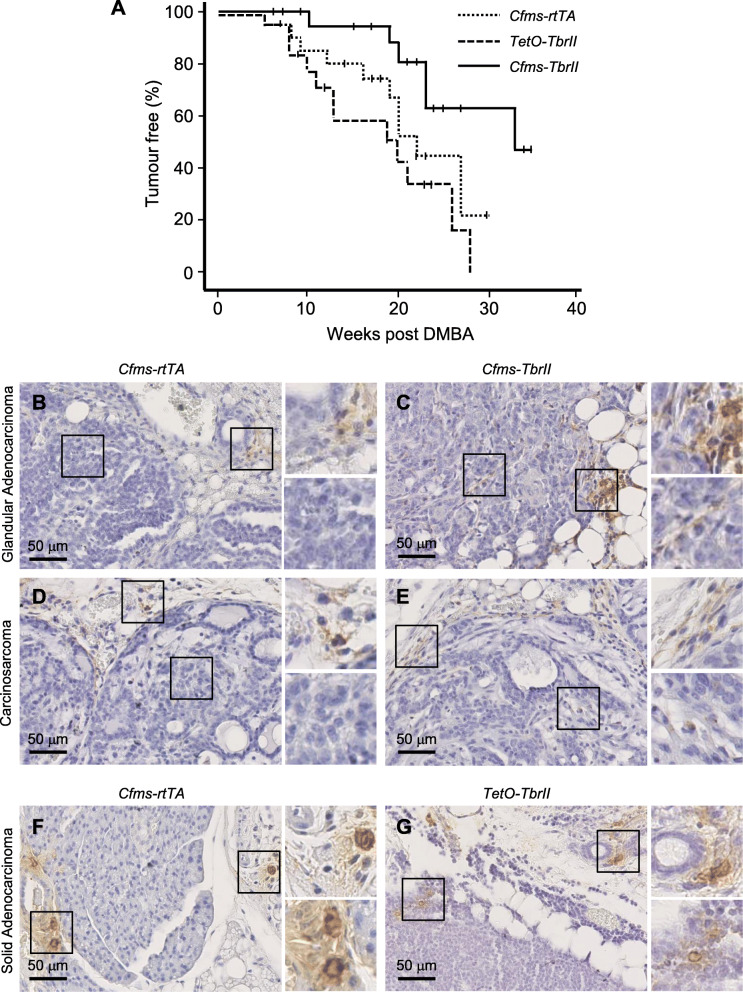
The effect of attenuated TGFB signalling in macrophages on mammary gland cancer susceptibility in mice. Mammary tumour latency was monitored following DMBA administration in doxycycline-treated *Cfms-rtTA*, *TetO-TbrII* and *Cfms-TbrII* mice (*n* = 19–20/gp). Mice were palpated weekly, commencing 1 week after the final DMBA dose, with the zero time point representing mice 12 weeks of age. Tumour latency was determined as the time to the first appearance of palpable mammary tumour. The censored mice (mice were killed because of other tumours or sickness) were represented by a vertical line on the plot. Data are presented as Kaplan-Meier tumour-free survival plot (**a**). Significant difference observed by log rank analysis (*Cfms-rtTA* vs. *Cfms-TbrII*; *p* < 0.05 and *TetO-TbrII* vs. *Cfms-TbrII*; *p* < 0.05). Examples of F4/80 (**b**–**e**) and CCR7 (**f**, **g**) staining in tumours classified as glandular adenocarcinoma (**b**, **c**), carcinosarcoma (**d**, **e**) and solid adenocarcinoma (**f**, **g**), in tumour samples from *Cfms-rtTA* (**b**, **d**, **f**), *TetO-TbrII* (**g**) and *Cfms-TbrII* (**c**, **e**) mice

Doxycycline-treated *Cfms-rtTA*, *TetO-TbrII* and *Cfms-TbrII* mice were challenged with the chemical carcinogen DMBA to investigate mammary cancer susceptibility. Fewer DMBA-treated *Cfms-TbrII* mice developed mammary gland tumours [6 of 20 mice; 30%] compared to *Cfms-rtTA* [10 of 20 mice; 50%] and *TetO-TbrII* [12 of 19 mice; 63%] mice. Moreover, there was a significant increase in tumour-free survival in DMBA-treated *Cfms-TbrII* mice compared to *Cfms-rtTA* (*p* = 0.02) and *TetO-TbrII* (*p* = 0.004) mice (log rank analysis; Fig. 7a). No significant difference was observed in tumour-free survival between the two control groups.

Oral administration of DMBA carcinogen resulted in the development of heterogeneous tumour types within the mammary gland consistent with previous reports [30, 37]. Histologically, the tumours were classified as adenocarcinoma (solid and glandular), carcinosarcoma, adenosquamous carcinoma and adenoma/mammary intraepithelial neoplasia (MIN) (Table 2). F4/80-positive cells were detected surrounding all tumours, often observed as small clusters (Fig. 7b–e). CCR7-positive staining was also detected in the tissue surrounding the tumours (Fig. 7f, g). In some of the tissue samples, F4/80- and CCR7-positive staining was observed within the tumours. When this was observed, the staining was scattered and of lower abundance compared to staining in adjacent normal mammary tissue. F4/80- and CCR7-positive staining within tumours was scored as either none/minimal or few/scattered. Four of 10 *Cfms-rtTA* mice, 3 of 12 *TetO-TbrII* mice and 3 of 6 *Cfms-TbrII* mice exhibited scattered F4/80-positive macrophages (Fig. 7; Table 2). CCR7-positive staining was observed in the tumours of 2 of 10, 1 of 12, and 0 of 6 *Cfms-rtTA*, *TetO-TbrII* and *Cfms-TbrII* mice, respectively. This data was not statistically analysed due to the mixed histology of the tumours and the small sample size available. Staining for iNOS was not done as all the tumours were formalin-fixed and paraffin-embedded, and this tissue preparation technique is not compatible with iNOS immunohistochemistry.

**Table 2:** Assessment of histological tumour type and F4/80- and CCR7-positive staining of DMBA-induced tumours in doxycycline-treated *Cfms-rtTA*, *TetO-TbrII* and *Cfms-TbrII* mice. Histology was assessed in H&E-stained tissue sections and classified as adenocarcinoma (solid or glandular), carcinosarcoma, adenosquamous carcinoma and adenoma/mammary intraepithelial neoplasia (MIN). F4/80 and CCR7 immunohistochmistry staining was classified as either none/minimal or scattered. The number of tumours of each type for each genotype is presented, and the percent and the number of those tumours exhibiting F4/80- or CCR7-positive staining classified as scattered are shown. In some cases, tumour staining was not assessed (N/A) as there was no tumour present

	*Cfms-rtTA*		*TetO-TgfbrII*		*Cfms-TbrII*	
	F4/80	CCR7	F4/80	CCR7	F4/80	CCR7
Adenocarcinoma (solid)	3		5		0	
% (*N*)	33 (1)	67 (2)	0 (0)	20 (1)	N/A	N/A
Adenocarcinoma (glandular)	1		0		4	
% (*N*)	0 (0)	0 (0)	N/A	N/A	50 (2)	0 (0)
Carcinosarcoma	2		3		2	
% (*N*)	0 (0)	0 (0)	0 (0)	0 (0)	50 (1)	0 (0)
Adenosquamous carcinoma	3		3		0	
% (*N*)	67 (2)	0 (0)	67 (2)	0 (0)	N/A	N/A
Adenoma/MIN	1		1		0	
% (*N*)	100 (1)	0 (0)	100 (1)	0 (0)	N/A	N/A
Total	10		12		6	
% (*N*)	40 (4)	20 (2)	25 (3)	8 (1)	50 (3)	0 (0)

### Inverse relationship between epithelial cell-associated TGFB1 and macrophage abundance in human non-neoplastic breast tissue

**Fig. 8:**
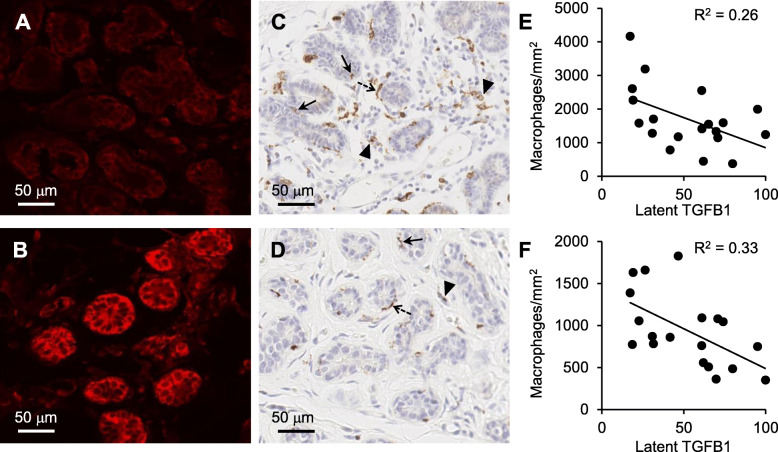
Relationship between latent TFGB1 and the abundance of CD68-positive macrophages in human breast tissue. Representative latent TGFB1 (**a**, **b**) and CD68 (**c**, **d**) antibody staining of human non-neoplastic breast tissue. Examples are of high (**a**) and low (**b**) staining intensity for latent TGFB1 and the same patient sample stained with CD68 antibody (**c** and **d,** respectively). Epithelial-associated CD68-positive macrophages were located between adjacent epithelial cells (arrows), epithelial-aligned CD68-positive macrophages were macrophages that aligned along the epithelium but not invaded into the epithelium (dashed arrows), and stromal CD68-positive macrophages were macrophages within the stroma (arrowheads). The intensity of latent TGFB1 staining and abundance of CD68-positive macrophages in breast tissue was analysed by linear regression (*n* = 19); latent TGFB1 vs. CD68-positive macrophages in the stroma, *p* < 0.05 (**e**); latent TGFB1 vs. CD68-positive macrophages associated with epithelium, *p* < 0.05 (**f**)

Having shown that a key role for TGFB in the mouse mammary gland is regulation of macrophages, with an impact on DMBA-induced tumour susceptibility, we next explored whether TGFB affects macrophage populations in human breast tissue. Latent TGFB1 was observed to be largely associated with the mammary epithelium; however, the abundance of TGFB1 was varied amongst participants and ranged from low to high expression (Fig. 8a, b). No positive staining was observed in human breast stained with isotype-matched irrelevant antibody (Supplementary Figure S3).

CD68 is a pan macrophage marker which is highly expressed by human monocytes and tissue macrophages [38]. CD68-positive cells were found to be located within three different locations: (i) epithelial-associated CD68-positive macrophages were located between adjacent epithelial cells, (ii) epithelial-aligned CD68-positive macrophages were macrophages that aligned along the epithelium but not invaded into the epithelium and (iii) stromal CD68-positive macrophages were macrophages within the stroma (Fig. 8c, d). No positive staining was observed in human breast stained with isotype-matched irrelevant antibody (Supplementary Figure S2D). There was no statistical correlation between the latent TGFB1 expression and the abundance of epithelial-aligned CD68-positive macrophages (data not shown). However, there was a significant inverse relationship between the abundance of latent TGFB1 and the abundance of both epithelial-associated macrophages (Fig. 8e; *R*^2^ = 0.26, *p* = 0.27) and stromal macrophages (Fig. 8f; *R*^2^ = 0.33, *p* = 0.01). No statistical correlation was observed between macrophage abundance and latent TGFB1 with patient characteristics such as age or menopausal status.

## Discussion

TGFB is one of the key signalling pathways that regulates mammary gland development [39, 40, 41]. Many studies have demonstrated that TGFB signalling can inhibit mammary gland development in an autocrine manner [40, 42, 43], whereby diminished synthesis or signalling capacity of TGFB1 results in increased ductal elongation. Further transplantation studies have revealed that TGFB1 has both autocrine and paracrine effects on mammary gland development as mice with a deficiency in epithelial cell-derived TGFB1 exhibited an overall normal mammary gland development, despite an increased rate of epithelial cell turnover [21] and accelerated ductal elongation [11]. Our observation of a small increase in the alveolar epithelium in mice with attenuated TGFB signalling in macrophages suggests a feedback loop between epithelial cells and local macrophages occurs, whereby epithelial cell-derived TGFB1 acts on macrophages that in turn regulate mammary epithelial cell development.

Macrophages play diverse and essential roles in mammary gland development [15–17], under the direction of cytokine signalling derived from the local tissue microenvironment [44]. In response to various signals, macrophages can undergo classical “M1” activation or alternative “M2” activation [34]. The “M1” phenotype is characterised by the expression of pro-inflammatory cytokines, high production of reactive oxygen and nitrogen intermediates which can promote tumour suppression. In contrast, “M2” macrophages are considered to be involved in tissue remodelling and promoting tumour progression [34, 45]. For macrophages to fulfil multiple functions during mammary gland development and cancer progression, they function under the direction of different cytokines and hormones. TGFB1 regulates macrophage function in many tissues, and therefore, TGFB signalling could be one of the key signalling pathways involved in maintaining the balance of different populations of macrophages in the cycling mammary gland, enabling the mammary gland to undergo appropriate development and regression over the course of each ovarian cycle.

Doxycycline is a commonly prescribed antibiotic and is used experimentally in conditional transgenic studies. Doxycycline is also known to regulate macrophage polarisation and promotes a shift toward an “M1” type macrophage cell type in vitro and in choroidal tissue when administered intraperitoneally to mice [46]. It is possible in the present study that mammary macrophages were shifted toward the “M1” type macrophage phenotype due to doxycycline provided in drinking water from the age of 6 weeks. As all the mice described in these studies were similarly treated, the differences observed between single transgenic controls and the double transgenic *Cfms-TbrII* mice are the result of TGFB attenuation in the transgenic model, rather than doxycycline treatment.

Epithelial cell-derived-TGFB1 has previously been demonstrated to prevent macrophage invasion into the mammary epithelium and suppress the activities of inflammatory type “M1” macrophages [21]. In agreement with this, we found an increased abundance of macrophages in both the ductal and alveolar epithelium in the mammary glands with attenuated TGFB signalling in macrophages, suggesting that TGFB signalling plays a significant role in regulating macrophage abundance and invasion. The macrophages observed within the mammary epithelium were identified by F4/80 staining and did not express either iNOS or CCR7. Macrophage populations in the mammary gland are highly heterogeneous and can be defined by distinct markers and tissue location. Ductal epithelium-associated macrophages express MHCII, CD11b and CD11c; exhibit long dendrite projections; and are intercalated between the epithelial bilayers of the duct [47, 48]. This population of macrophages express a gene signature that resembles tumour-associated macrophages [48].

In addition, impaired TGFB signalling in macrophages caused an increased abundance of “M1” iNOS-positive and CCR7-positive macrophages within the mammary gland stroma. Our findings are further supported by studies which have demonstrated that TGFB1 inhibits the expression of macrophage scavenger receptor during macrophage-mediated phagocytosis and downregulates the expression of “M1” marker iNOS in activated macrophages, thus resolving inflammation and preventing disease progression [49–53]. Taken together, this evidence strongly suggests the role of TGFB signalling to macrophages in the mammary gland appears to be twofold, through (1) inhibition of macrophage invasion into the epithelium and (2) downregulation of “M1” macrophages.

TGFB1 has both stimulatory and inhibitory roles in cancer progression; it acts as a tumour suppressor at the early stage of cancer development and promotes invasion and metastasis at the later stage of cancer progression [12–14]. The increased risk of breast cancer conferred by the presence of the TGFB1 L10P gene polymorphism suggests that increased production of TGFB1 is associated with increased susceptibility to breast cancer [22]. This is consistent with our observations, as a significant reduction in the incidence of mammary gland tumour formation, and longer tumour latency was observed in mice with attenuated TGFB signalling in macrophages challenged with DMBA carcinogen, indicating that TGFB-regulated macrophages might promote mammary cancer susceptibility in mice.

The balance of pro-tumourigenic or anti-tumourigenic activities of macrophages is dependent on cytokine signals derived from the local tissue microenvironment, including TGFB signalling [44, 54]. Consequently, attenuated TGFB signalling may alter the phenotypes and functions of macrophages and thus affect cancer susceptibility. The reduction of mammary gland tumour incidence in mice with impaired TGFB signalling in macrophages could be due to the increased abundance of nitric oxide-producing macrophages with enhanced capacity for immune surveillance [55]. Thus, aberrant TGFB signalling to macrophages may impair immune system protection from tumour formation leading to an increased risk of breast cancer. Conclusions on the role of TGFB-regulated macrophages on the further development of tumours, beyond tumour initiation induced by DMBA, was not possible. Differences in the distribution and phenotype of macrophages were observed between TGFB-attenuated and control mice, but this could be due to the variable histological tumour types observed.

Similar to the mouse mammary gland, TGFB1 is observed in human non-neoplastic breast epithelium; however, the expression level was found to vary between individuals. Macrophages are mainly located around the epithelium and within the epithelium and the stroma, and therefore are in close proximity to TGFB1. The significant inverse relationship between the abundance of TGFB1 and the abundance of macrophages is in agreement with our finding that reduced TGFB signalling increases macrophage abundance in the double transgenic mouse model. Taken together, this suggests that epithelial cell-derived TGFB1 is likely to exert regulatory actions on macrophages in the human breast, to an extent that varies between individuals dependent on the abundance and bioavailability of the cytokine.

## Conclusion

TGFB1 has a significant role in regulating macrophages in the mammary gland and impacts upon normal development and susceptibility to mammary cancer. We propose that TGFB signalling in macrophages promotes mammary gland tumorigenesis in mice through suppression of immune surveillance activities of macrophages. Given the diverse nature of TGFB1 action, it is not desirable to broadly target this cytokine as a therapeutic strategy. However, a better understanding of the precise cell types and signalling pathways that result in the tumour-promoting effects of TGFB1 could lead to new approaches for breast cancer prevention in the future.

## Supplementary Information

**Additional file 1: Supplementary Figure 1.** Representative images for single-stained controls for immunofluorescence of F4/80 (A-C) and pSMAD2 (D-E), showing F4/80 (red channel; A, D), pSMAD2 (green channel; B, E), and merged images showing co-localisation and DAPI (blue channel) nuclear stain (C, F).

**Additional file 2: Supplementary Figure 2.** Representative images for isotype controls for F4/80 (A), CCR7 (B), iNOS (C), and CD68 (D) immunohistochemistry in mouse mammary gland (A-C) and human non-neoplastic breast tissue (D).

**Additional file 3: Supplementary Figure 3.** Detection of latent TGFB1 in human non-neoplastic breast tissue. Anti-latent TGFB was used to detect TGFB1 expression in non-neoplastic human breast tissue (*n*=19) (A and B), and negative control of secondary antibody only (C). The sections were also counterstained with DAPI (D, E and F). Latent TGFB1 and DAPI were visualised simultaneously under a confocal microscope (G: merged picture of A and D; H: merged picture of B and E; I: merged picture of C and F). Immunostainings of A, D and G represented breast tissue with low level expression of TGFB1, whereas immunostainings of B, E and H represented breast tissue with high level expression of TGFB1.The application of secondary antibodies only on human breast tissue (C, F, and I) was used as negative control.

## Data Availability

The datasets used and/or analysed during the current study are available from the corresponding author on reasonable request.
