# Supplementary figures and images for: Attenuated TGFB signalling in macrophages decreases susceptibility to DMBA-induced mammary cancer in mice
Source: Breast Cancer Res. 2021 Mar 24;23:39. doi: 10.1186/s13058-021-01417-8 (PMC7992865; doi:10.1186/s13058-021-01417-8)

## Slide 1
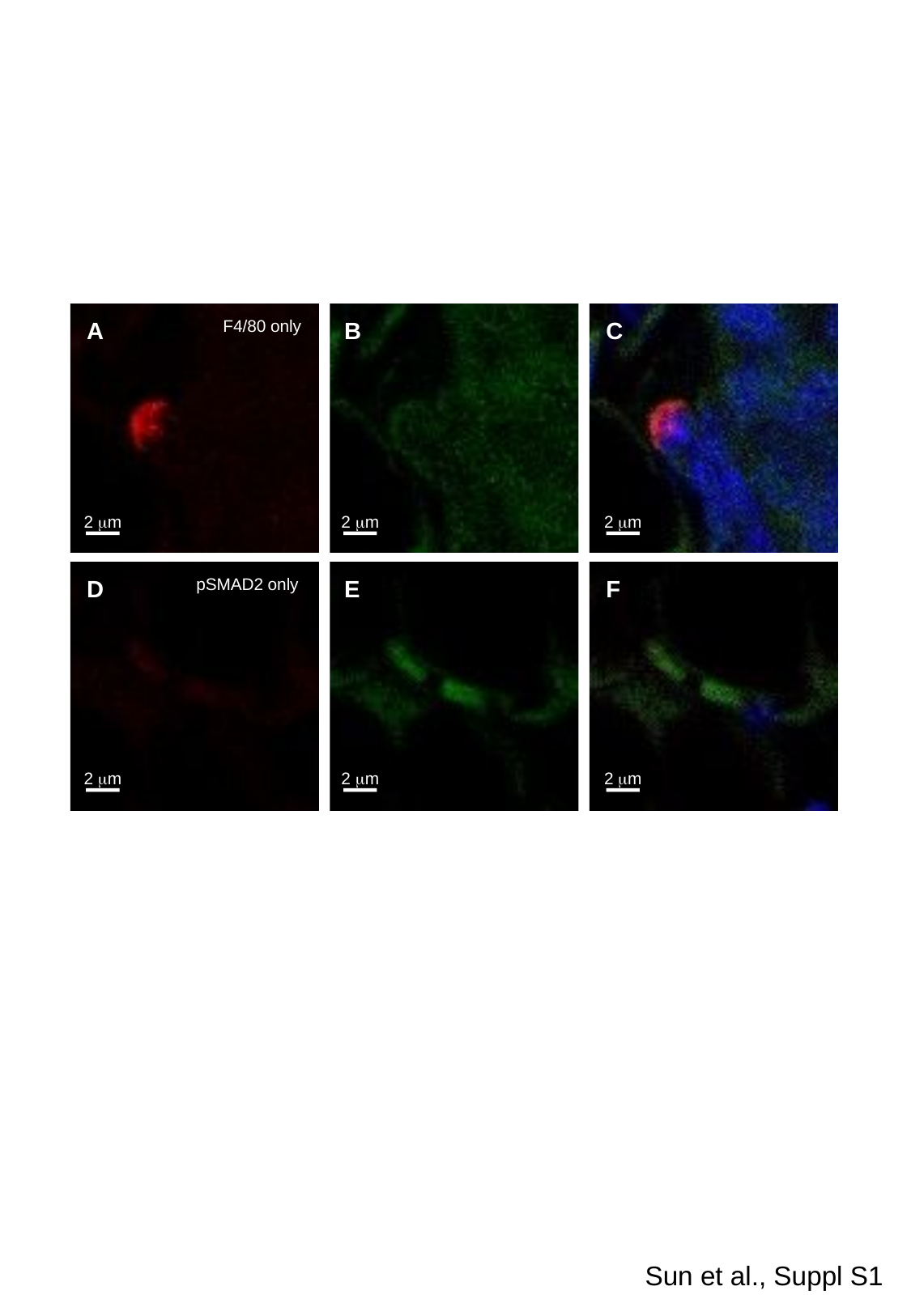

A
F4/80 only
B
C
2 mm
2 mm
2 mm
D
pSMAD2 only
E
F
2 mm
2 mm
2 mm
Sun et al., Suppl S1

Supplement: Supplementary file 1 — Additional file 1: Supplementary Figure 1. Representative images for single-stained controls for immunofluorescence of F4/80 (A-C) and pSMAD2 (D-E), showing F4/80 (red channel; A, D), pSMAD2 (green channel; B, E), and merged images showing co-localisation and DAPI (blue channel) nuclear stain (C, F). [file 13058_2021_1417_MOESM1_ESM.pptx]

## Slide 1
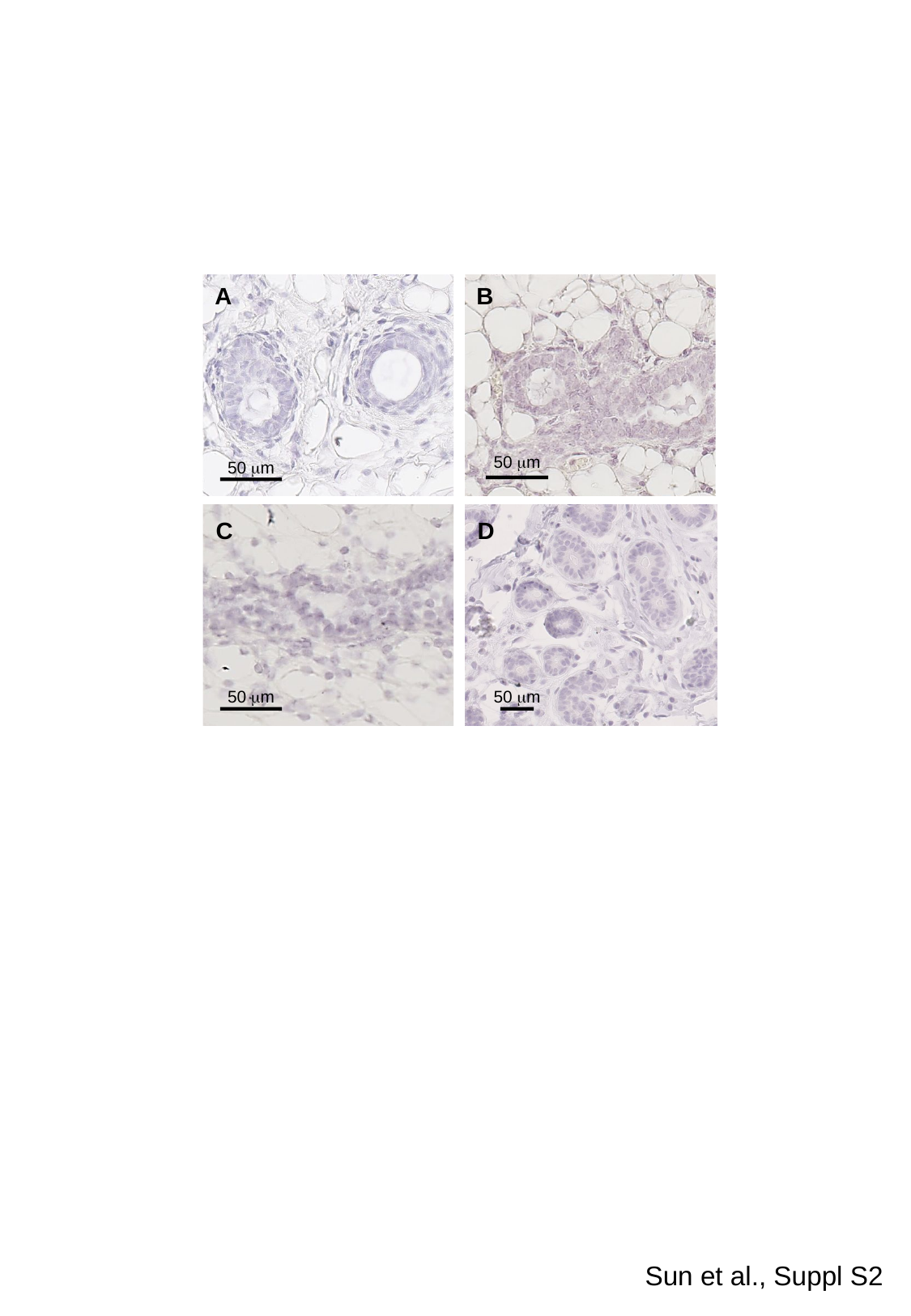

A
B
50 mm
50 mm
C
D
50 mm
50 mm
Sun et al., Suppl S2

Supplement: Supplementary file 2 — Additional file 2: Supplementary Figure 2. Representative images for isotype controls for F4/80 (A), CCR7 (B), iNOS (C), and CD68 (D) immunohistochemistry in mouse mammary gland (A-C) and human non-neoplastic breast tissue (D). [file 13058_2021_1417_MOESM2_ESM.pptx]
