# Supplementary material for: Attenuated TGFB signalling in macrophages decreases susceptibility to DMBA-induced mammary cancer in mice
Source: Breast Cancer Res. 2021 Mar 24;23:39. doi: 10.1186/s13058-021-01417-8 (PMC7992865; doi:10.1186/s13058-021-01417-8)
Supplement: Supplementary file 3 — Additional file 3: Supplementary Figure 3. Detection of latent TGFB1 in human non-neoplastic breast tissue. Anti-latent TGFB was used to detect TGFB1 expression in non-neoplastic human breast tissue (n=19) (A and B), and negative control of secondary antibody only (C). The sections were also counterstained with DAPI (D, E and F). Latent TGFB1 and DAPI were visualised simultaneously under a confocal microscope (G: merged picture of A and D; H: merged picture of B and E; I: merged picture of C and F). Immunostainings of A, D and G represented breast tissue with low level expression of TGFB1, whereas immunostainings of B, E and H represented breast tissue with high level expression of TGFB1.The application of secondary antibodies only on human breast tissue (C, F, and I) was used as negative control. [file 13058_2021_1417_MOESM3_ESM.pptx]

## Slide 1
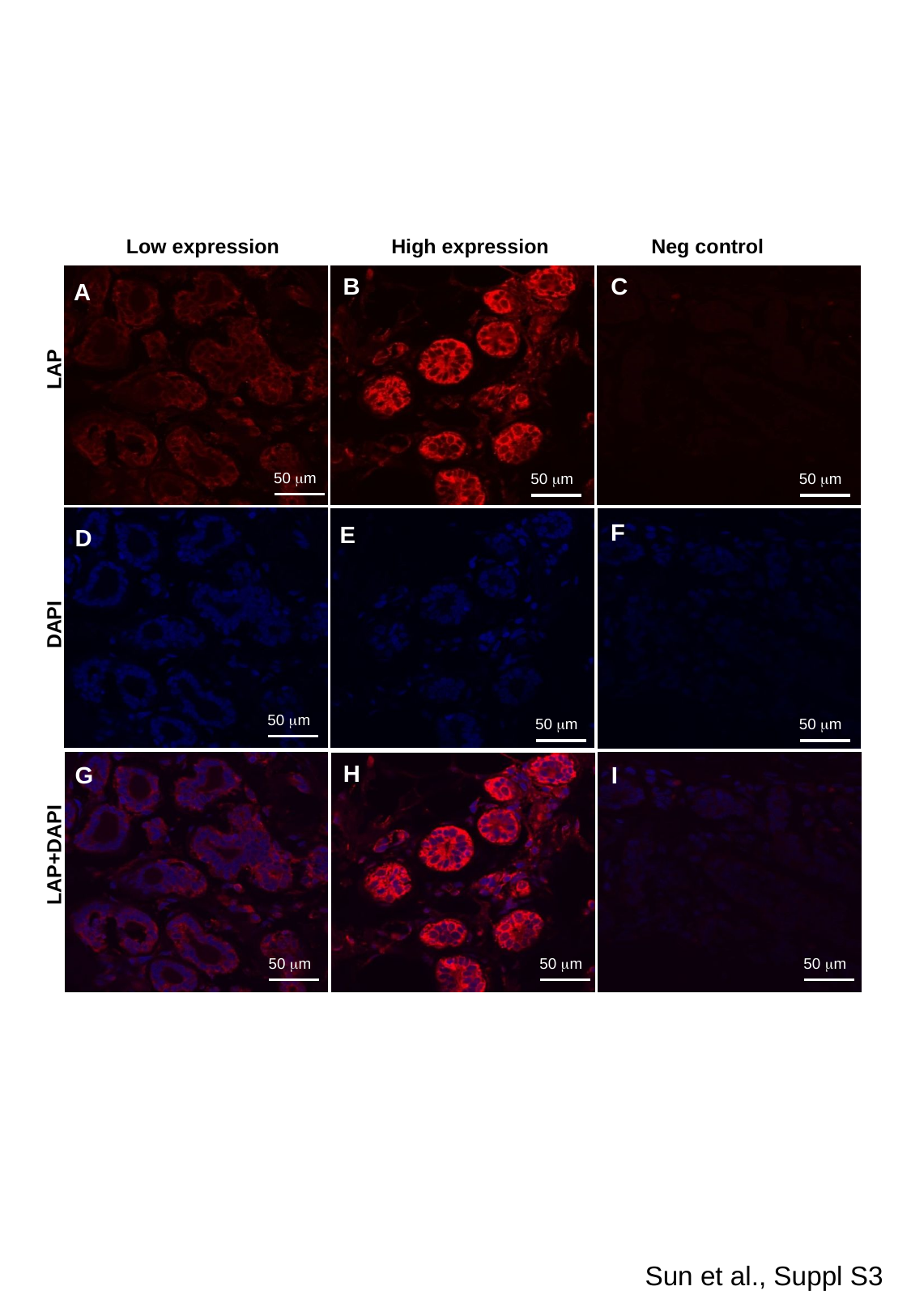

Neg control
Low expression
High expression
B
C
A
F
E
D
H
G
I
50 mm
50 mm
50 mm
50 mm
50 mm
50 mm
50 mm
50 mm
50 mm
LAP
DAPI
LAP+DAPI
Sun et al., Suppl S3
